# Can Artificial Intelligence Support Patient Education in Scabies? A Comparative Analysis of Large Language Model Responses

**DOI:** 10.3390/healthcare14101278

**Published:** 2026-05-08

**Authors:** Mahmut Talha Uçar, Ecem Bostan, Elif Dönmez, Fatma Cerit Soydan

**Affiliations:** 1 Saray District Health Directorate, Tekirdağ Provincial Health Directorate, Ministry of Health, Tekirdağ 59100, Türkiye; mahmuttalha.ucar@saglik.gov.tr; 2 Dermatology and Venereology Department, Faculty of Medicine, Ankara Medipol University, Ankara 06050, Türkiye; ecem.bostan@ankaramedipol.edu.tr; 3 Oncology Nursing Department, Hamidiye Faculty of Nursing, University of Health Sciences, Istanbul 34668, Türkiye; 4 Department of Nursing, Faculty of Health Sciences, Istanbul Topkapi University, Istanbul 34087, Türkiye; fatmaceritsoydan@topkapi.edu.tr

**Keywords:** scabies, artificial intelligence, chatbots, patient education, dermatology, readability

## Abstract

**Introduction:** Artificial intelligence (AI)-based chatbots are becoming an increasingly popular source of health information, particularly for common dermatological conditions such as scabies. However, concerns remain about the accuracy, reliability, quality and readability of the information they provide. **Objectives:** The aim of this study was to evaluate the accuracy, reliability, quality and readability of responses generated by different AI chatbots in answer to patient questions about scabies. **Methods:** Scabies-related questions were collected from Quora, a publicly accessible question-and-answer platform, and screened for relevance. Following expert review, 20 representative questions were selected. Responses were generated by three large language models: ChatGPT-5.2, DeepSeek and Claude Sonnet 4.5. The outputs were evaluated by expert reviewers using the hallucination rate, modified DISCERN (mDISCERN), Global Quality Score (GQS), Flesch Reading Ease Score (FRES), and an accuracy assessment based on a 5-point Likert scale. **Results:** In this study, it was found that ChatGPT-5.2 demonstrated the highest information quality (mDISCERN: 33.6 ± 1.8) and readability (FRES: 63.25 ± 11.5). DeepSeek achieved the highest global quality score (GQS: 5.00 ± 0.00) and accuracy score (5.00 ± 0.00). Claude Sonnet 4.5 had lower scores across most metrics. There were significant differences in hallucination rates among the models (*p* = 0.003), with DeepSeek exhibiting higher rates. Overall, statistically significant differences were observed among the models in terms of quality, readability and accuracy. **Conclusions:** AI chatbots provide generally informative but variable-quality responses to scabies-related questions. While DeepSeek demonstrated higher accuracy and overall quality, it also showed higher hallucination rates, whereas ChatGPT-5.2 provided more readable and reliable responses. These findings highlight variability across models and the need for cautious use. AI tools should be considered supportive resources rather than substitutes for professional medical advice.

## 1. Introduction

Artificial intelligence (AI) is a technology that aims to replicate cognitive processes requiring human intelligence—such as perception, learning, decision-making, and natural language processing—through computer systems, and it is increasingly being used in healthcare to improve diagnostic accuracy, strengthen clinical decision-support systems, support patient education, and facilitate access to health services [1]. Dermatology, a discipline highly dependent on visual assessment, clinical reasoning, and patient–physician interaction, has become one of the medical fields in which AI applications have been rapidly adopted. In particular, large language models such as ChatGPT have begun to be utilized in dermatology for patient education, responding to questions related to common skin diseases, providing preliminary information in teledermatology settings, supporting clinical decision-making processes, assisting in preparation for specialty examinations, and contributing to medical writing [2]. However, studies evaluating ChatGPT’s performance on dermatology specialty examinations and clinical questions indicate that, although the model shows promise in terms of knowledge provision and patient information, it demonstrates limited accuracy in clinical procedures and complex diagnostic processes when compared with human experts [3]. Therefore, the existing literature emphasizes that AI should be regarded not as a substitute for dermatologists, but rather as a complementary tool that supports clinical decision-making processes and enhances patient care in dermatological practice [1,2].

Scabies is a parasitic skin disease with a variable yet often high prevalence worldwide; although regional differences exist, recent meta-analyses report a substantial global average prevalence [4,5]. The clinical management of scabies is challenging due to diagnostic difficulties, rapid transmission among patients, shortcomings in treatment implementation or drug supply, the risk of secondary bacterial infections, and the presence of treatment-resistant forms, including chronic and crusted scabies, all of which contribute to a notable public health burden [6,7]. Scabies was chosen as a model condition for several reasons. It is highly prevalent, presents diagnostic challenges, and carries substantial public health consequences. In addition, patient education plays a crucial role in both preventing the spread of the disease and ensuring that affected individuals receive appropriate treatment. In recent years, digital health tools—particularly symptom-checker chatbots and AI-supported mobile applications—have become increasingly widespread, enabling the public to rapidly query symptoms and access basic health information. In the study conducted by Podder et al. [8], chatbots were shown to be used for preliminary screening and patient education in dermatological diseases. Recent developments such as AI-based diagnostic tools (e.g., ScabAI) further illustrate the expanding role of AI in scabies management [9]. However, the performance of different AI systems varies. Large language models and conversational AI tools show promise in patient education and answering frequently asked dermatological questions; however, empirical evaluations reveal variability in model performance and limitations in complex, clinically nuanced, and image-intensive cases [1,2,3].

Scabies remains a highly prevalent and clinically challenging dermatological condition, characterized by delayed diagnosis, high transmissibility, and substantial impacts on individual and public health outcomes. In parallel, artificial intelligence–based chatbots have become increasingly used by patients as readily accessible sources of medical information, particularly for common and stigmatized skin conditions. Despite their growing popularity, there is limited empirical evidence specifically evaluating large language models in scabies-related patient education. Given the potential risks of misinformation, inappropriate self-management, delayed medical consultation, and potential public health consequences, a systematic evaluation of chatbot-generated responses is needed. Therefore, this study aims to evaluate the accuracy, reliability, quality, and readability of responses generated by artificial intelligence chatbots to scabies-related questions, addressing an important gap in the current dermatology and digital health literature. Furthermore, this study adds to the existing literature by offering a disease-specific, multi-dimensional assessment of large language models in the context of scabies patient education. It does so by evaluating not only content quality but also citation reliability, using real-world questions generated by patients themselves.

## 2. Materials and Methods

### 2.1. Data Source and Question Selection

Patient-generated questions were collected from Quora, a publicly accessible question-and-answer platform, due to its explicit question-based structure, which enables systematic identification of health-related information needs expressed in everyday language. Similar methodologies have been employed in prior dermatology-focused chatbot evaluations [10]. The platform was searched using the keyword “scabies”, and the “Questions” filter was applied to restrict results to user-initiated inquiries. The data collection was performed on 20 December 2025, and the search was conducted manually using the platform’s default interface without automated data extraction tools. To ensure a standardized and reproducible sampling frame, the first 200 questions returned by the platform’s default relevance-based ranking were systematically reviewed, with no time restriction applied. Quora’s ranking algorithm is based on multiple factors, including relevance, user engagement (e.g., views and interactions), and content quality. While this approach may highlight commonly discussed topics, the final selection of questions was determined through expert evaluation to ensure clinical relevance and generalizability.

### 2.2. Screening and Eligibility Criteria

An initial screening was conducted to exclude questions that were not relevant to human scabies or clinical dermatology. Questions were removed prior to screening if they met any of the following criteria: i. animal-related or veterinary scabies (e.g., dogs or livestock), or ii. non-medical or unrelated content, including historical, speculative, or non-clinical discussions.

The remaining questions were then screened based on relevance and generalizability. During this stage, questions were excluded if they were: i. duplicate or near-duplicate versions of previously identified questions, or ii. highly individualized or case-specific inquiries, defined as questions containing detailed personal medical histories, image-based descriptions, or patient-specific contexts (e.g., “Is this lesion on my skin scabies?”) that limit generalizability to routine clinical practice.

These exclusion criteria were applied to ensure that the selected questions reflected generalizable patient concerns rather than individual clinical cases.

### 2.3. Expert Review and Final Selection

Following the screening process, 174 questions remained after the exclusion of non-relevant and duplicate content. From this pool, 40 unique and representative questions were selected for expert evaluation based on relevance and thematic diversity.

These 40 questions were independently reviewed by a board-certified dermatologist and a public health specialist to assess clinical relevance, generalizability, and thematic coverage. Questions were excluded after expert review if they: i. provided limited additional clinical relevance beyond existing questions, or ii. demonstrated substantial conceptual overlap with other shortlisted questions.

**Figure 1:**
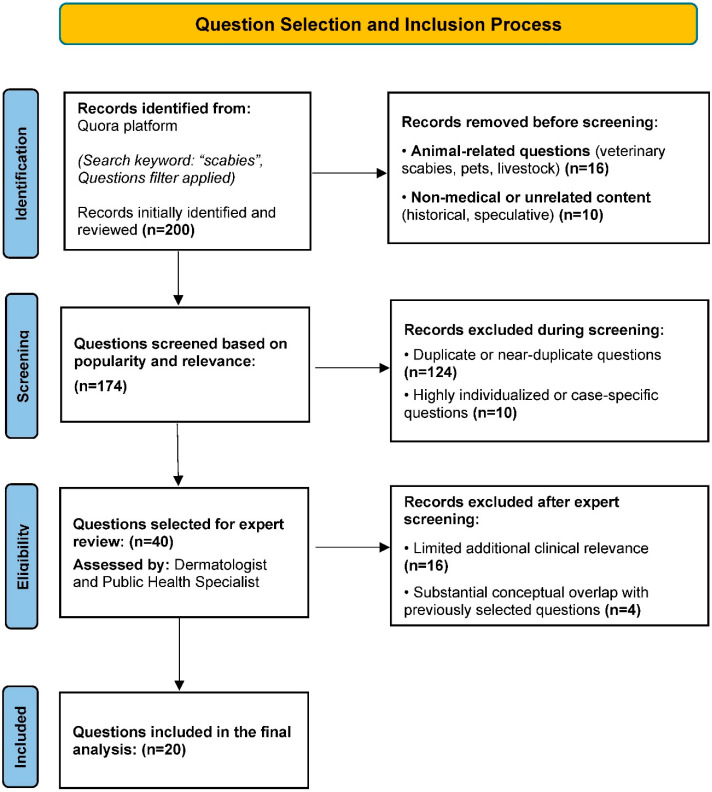
PRISMA-based flow diagram illustrating the identification, screening, eligibility assessment, and inclusion of scabies-related questions retrieved from the Quora platform.

Following this evaluation, the final set of 20 questions was determined through consensus-based discussion between the two researchers. The final number of questions was limited to 20 to allow detailed expert-based evaluation across multiple assessment domains. These questions were selected to represent common and generalizable patient concerns encountered in routine clinical practice, including disease transmission, diagnosis, symptoms, treatment options, environmental decontamination, recurrence, and post-treatment outcomes. A detailed overview of the question identification, screening, eligibility assessment, and final inclusion process is presented in Figure 1.

This study obtained AI-generated responses from three large language models, each representing different architectural and communicative approaches: ChatGPT-5.2, DeepSeek, and Claude Sonnet 4.5. These models were chosen for several reasons. First, they are widely used. Second, they differ in their underlying architectures and how they generate responses. Third, each has relevance to health-related information delivery. ChatGPT is a general-purpose model with broad adoption. DeepSeek has drawn attention for its strong reasoning abilities. Claude, in turn, has been recognized for producing structured and safety-conscious responses, especially in knowledge-intensive fields.

AI-generated responses were obtained between 21 and 22 December 2025 using publicly available web interfaces of ChatGPT (OpenAI, https://chat.openai.com, accessed on 22 December 2025), DeepSeek (https://www.deepseek.com, accessed on 22 December 2025), and Claude Sonnet (Anthropic, https://claude.ai, accessed on 22 December 2025). All models were accessed via their publicly available web interfaces without modification of default parameters. For each question, a new session was initiated in each chatbot to avoid context carryover effects, and the same question was presented identically across all models to ensure consistency. Each question was entered once for each model. After the initial response, a standardized follow-up prompt was used to request full bibliographic citations: “For the information provided above, please provide the full bibliographic citations (including authors, publication year, title, and journal/source) for the specific external references used. If applicable, provide the direct URL link in plain text as well.” The citation responses were recorded separately alongside the initial responses for analysis. All AI-generated responses and citation outputs were recorded and are provided in the Supplementary Materials.

### 2.4. Evaluation Instruments

#### 2.4.1. Reliability Assessment (Modified DISCERN)

The dermatologist used the modified DISCERN instrument to evaluate each response’s reliability. This instrument consists of eight items that assess the following: clarity of aims, achievement of objectives, relevance, citation of sources, timing of publication, balance and impartiality, provision of supplementary resources, and acknowledgement of uncertainty. Total scores ranged from 8 to 40, with each item being graded on a 5-point Likert scale (1 being low and 5 being high). Greater information integrity and dependability are indicated by higher ratings [11].

#### 2.4.2. Quality Assessment (Global Quality Scale—GQS)

The Global Quality Scale (GQS), a validated 5-point tool intended to evaluate the coherence, comprehensiveness, and patient-centered utility of online health information, was also used by the dermatologist to judge overall quality. A score of five suggested great content flow and significant patient benefit, whereas a score of one indicated poor quality and little value [10,12].

#### 2.4.3. Readability Assessment (Flesch Reading Ease Score—FRES)

Readability was evaluated using the Flesch Reading Ease Score (FRES), a widely used quantitative measure of textual readability originally developed by Flesch [13]. The FRES was calculated using a publicly available online readability calculator (https://readabilityformulas.com) [14]. Scores range from 0 to 100, with higher scores indicating easier readability. Standard classification thresholds were applied as follows: very easy (90–100), easy (80–89), fairly easy (70–79), standard (60–69), fairly difficult (50–59), difficult (30–49), and very difficult (0–29).

### 2.5. Accuracy Assessment

A standardized five-point Likert-type scale, modified from earlier research assessing big language model performance in medical information delivery, was used to evaluate the factual accuracy of each AI-generated response [10,15]. Recent research has frequently used this method to assess factual accuracy as well as conformity to accepted clinical norms.

The following criteria were used to assess the responses, which ranged from 1 to 5:

1 is completely false or contains misleading information; 2 is mostly false with few correct elements; 3 is a combination of accurate and inaccurate statements; 4 is mostly accurate with a few small errors or omissions; and 5 is completely accurate and in line with the most recent evidence-based dermatological guidelines.

Two evaluators with backgrounds in public health and dermatology conducted each evaluation on their own. Structured discussions were used to resolve any differences in ratings until an agreement was reached.

### 2.6. Assessment of Hallucination Rate

Hallucination was defined as fabricated or unverifiable references provided by the models rather than inaccuracies in the core informational content. The hallucination rate of AI-generated citations was evaluated by manually verifying all references provided by each model. Each cited reference was checked individually for accuracy, including title, first author, publication year, and source.

A reference was classified as “hallucinated” if it met any of the following criteria: i. the reference did not exist, or ii. at least two key bibliographic elements (title, first author, or publication year) were incorrect.

The hallucination rate was calculated as the proportion of hallucinated references divided by the total number of references provided by each model. Responses that did not include any references were recorded separately and analyzed descriptively.

All evaluations were conducted by designated researchers according to their areas of expertise to ensure methodological consistency. Reliability (mDISCERN), overall quality (GQS), and accuracy assessments were performed by the dermatologist (E.B.). Readability (FRES) was evaluated by the public health specialist (M.T.U.). The hallucination rate was assessed by a public health nursing specialist (E.D.) through manual verification of all cited references.

### 2.7. Statistical Analysis

All statistical analyses were performed using IBM SPSS Statistics (version 27). Data normality was assessed using the Shapiro–Wilk test, as well as skewness and kurtosis values. As the data did not meet normal distribution assumptions, nonparametric tests, including the Friedman test and Wilcoxon signed-rank test, were used for comparisons. Kendall’s coefficient of concordance (W) was calculated to assess the level of agreement between the chatbot models in terms of their performance scores. A *p*-value of <0.05 was considered statistically significant.

## 3. Results

### 3.1. Hallucination Rate

Claude Sonnet 4.5 did not provide references in 10 of 20 questions. Among responses that included references, citation hallucination rates ranged from 0% to 25%. ChatGPT-5.2 provided references in all questions, with no hallucinated references detected. DeepSeek did not provide references in 3 questions and showed citation hallucination rates of 16.7% and 20.0% in two individual responses.

The detailed per-question results are provided in the Supplementary Materials.

A Friedman test was conducted to compare hallucination rates across Claude Sonnet 4.5, ChatGPT-5.2, and DeepSeek. The analysis included only the questions for which complete data were available for all models (n = 9).

The results revealed a statistically significant difference in hallucination rates among the models (χ^2^(2) = 11.474, *p* = 0.003).

Mean rank values were highest for Claude Sonnet 4.5 (2.67), followed by DeepSeek (1.72) and ChatGPT-5.2 (1.61).

The effect size, as measured by Kendall’s W, was 0.637, indicating a strong level of agreement and a large effect size.

### 3.2. Reliability, Overall Quality, Readability, Accuracy

The descriptive statistics for reliability, overall quality, readability, and accuracy across the three models are summarized in Table 1.

### 3.3. Reliability

The modified DISCERN (mDISCERN) scores were compared among Claude Sonnet 4.5, ChatGPT-5.2, and DeepSeek across 20 scabies-related questions.

ChatGPT-5.2 had the highest mean mDISCERN score (33.60 ± 1.79), followed by DeepSeek (32.10 ± 2.13) and Claude Sonnet 4.5 (28.70 ± 4.16). Median values were higher for ChatGPT-5.2 and DeepSeek compared with Claude Sonnet 4.5. Claude Sonnet 4.5 showed a wider range and interquartile range.

A Friedman test revealed a statistically significant difference in mDISCERN scores among the three chatbot models (χ^2^(2) = 12.649, *p* = 0.002). Mean rank analysis showed that ChatGPT-5.2 had the highest rank (mean rank = 2.53), followed by DeepSeek (mean rank = 2.05), while Claude Sonnet 4.5 had the lowest rank (mean rank = 1.43). Kendall’s coefficient of concordance was 0.316, indicating the level of agreement among models.

Post hoc pairwise comparisons were conducted using the Wilcoxon signed-rank test with Bonferroni adjustment. ChatGPT-5.2 had significantly higher mDISCERN scores than Claude Sonnet 4.5 (adjusted *p* < 0.001). DeepSeek also had significantly higher scores than Claude Sonnet 4.5 (adjusted *p* = 0.012). No statistically significant difference was observed between ChatGPT-5.2 and DeepSeek (adjusted *p* = 0.075).

### 3.4. Overall Quality

The Global Quality Score (GQS) values differed across the three AI models. Descriptive analyses showed mean GQSs of 5.00 ± 0.00 for DeepSeek, 4.45 ± 0.51 for ChatGPT-5.2, and 4.10 ± 0.45 for Claude Sonnet 4.5.

A Friedman test revealed a statistically significant difference in GQSs among the three models (χ^2^(2) = 26.24, *p* < 0.001). Mean rank values were 2.70 for DeepSeek, 1.88 for ChatGPT-5.2, and 1.43 for Claude Sonnet 4.5. Kendall’s coefficient of concordance was 0.656, indicating the level of agreement among models.

Post hoc pairwise comparisons were conducted using the Wilcoxon signed-rank test with Bonferroni correction. DeepSeek had significantly higher GQSs than Claude Sonnet 4.5 (adjusted *p* < 0.001) and ChatGPT-5.2 (adjusted *p* = 0.027). ChatGPT-5.2 also had significantly higher GQSs than Claude Sonnet 4.5 (adjusted *p* = 0.020).

### 3.5. Readability

The readability of responses generated by Claude Sonnet 4.5, ChatGPT-5.2, and DeepSeek was assessed using the Flesch Reading Ease Score (FRES), where higher scores indicate easier readability.

The mean (±SD) FRES values were 63.25 ± 11.50 for ChatGPT-5.2, 51.30 ± 8.14 for DeepSeek, and 49.75 ± 10.93 for Claude Sonnet 4.5.

A Friedman test revealed a statistically significant difference in FRES distributions among the three models (χ^2^(2) = 21.949, *p* < 0.001). Kendall’s coefficient of concordance was 0.549.

Post hoc pairwise comparisons were conducted using the Wilcoxon signed-rank test with Bonferroni correction. ChatGPT-5.2 had significantly higher FRES values than Claude Sonnet 4.5 (adjusted *p* < 0.001) and DeepSeek (adjusted *p* < 0.001). No statistically significant difference was observed between Claude Sonnet 4.5 and DeepSeek (adjusted *p* = 0.559).

### 3.6. Accuracy

Accuracy scores of the three large language models were compared using descriptive statistics and nonparametric tests. The mean accuracy scores were 4.35 ± 0.49 for Claude Sonnet 4.5, 4.60 ± 0.50 for ChatGPT-5.2, and 5.00 ± 0.00 for DeepSeek.

A Friedman test revealed a statistically significant difference in accuracy scores among the three models (χ^2^(2) = 19.846, *p* < 0.001). Kendall’s coefficient of concordance was 0.496.

Post hoc pairwise comparisons with Bonferroni correction showed that DeepSeek had significantly higher accuracy scores than Claude Sonnet 4.5 (adjusted *p* = 0.006). No statistically significant differences were observed between ChatGPT-5.2 and Claude Sonnet 4.5 (adjusted *p* = 0.707) or between ChatGPT-5.2 and DeepSeek (adjusted *p* = 0.173).

**Figure 2:**
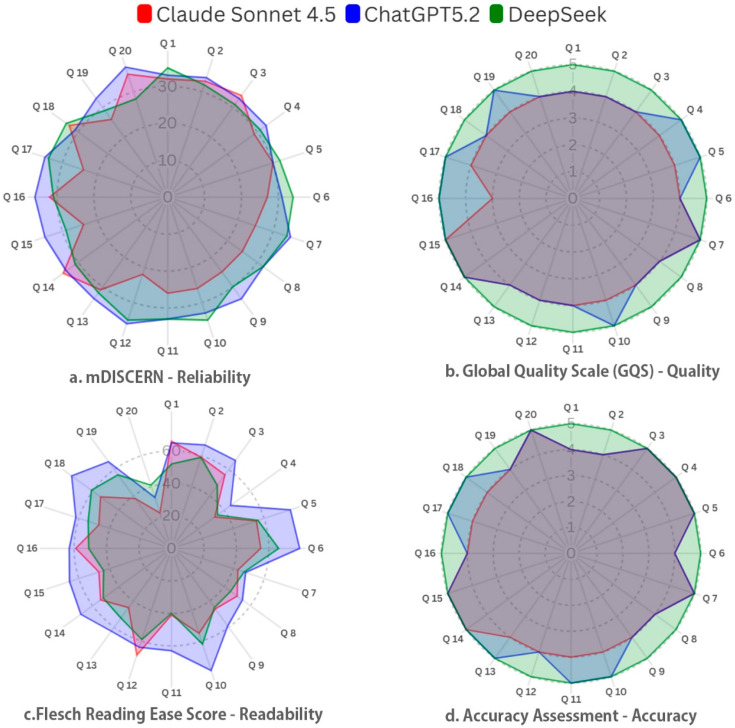
Radar plot comparison of item-level mDISCERN, GQS, FRES, and accuracy scores across 20 scabies-related questions.

**Figure 3:**
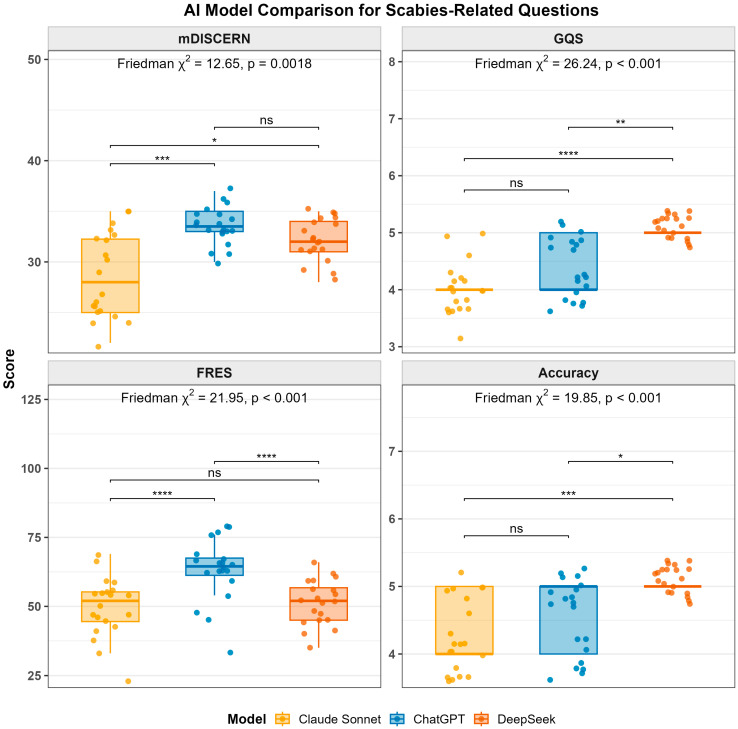
Boxplot distributions and post hoc pairwise comparisons of mDISCERN, GQS, FRES, and accuracy scores across AI models. Symbols *, **, ***, and **** indicate statistically significant differences between groups in pairwise comparisons based on Bonferroni-adjusted *p*-values (* *p* < 0.05, ** *p* < 0.01, *** *p* < 0.001, and **** *p* < 0.0001).

The distribution of item-level scores across the 20 questions is illustrated in Figure 2. The distribution of mDISCERN, GQS, FRES, and accuracy scores, along with post hoc pairwise comparisons, is illustrated in Figure 3.

## 4. Discussion

To our knowledge, this is the first study to systematically evaluate the performance of three different AI chatbots in providing scabies-related patient education. Even though the performances of only three generative AI chatbots are investigated in providing the responses to the most popular scabies-related questions, the present study provides noteworthy insight into the utility of AI for patient education. The outcomes show that different AI chatbots show distinctive results when evaluated in terms of quality, readability, credibility and hallucination rate. Across 20 scabies-related, most frequently asked questions, DeepSeek demonstrated higher quality and accuracy scores when compared to ChatGPT-5.2 and Claude Sonnet 4.5, whereas ChatGPT-5.2 had more comprehensible content in comparison to DeepSeek and Claude Sonnet-4.5. These heterogeneous, preliminary findings may suggest that different large language models have their own advantages and shortcomings, their efficiency may be enhanced when used in conjunction with clinical expertise.

Easy accessibility of the online content, has led individuals to resort to the traditional online search engines and AI tools for gathering information about various health conditions. The emergence of large language models which leverage cutting-edge AI technology and simulate human-to-human conversation, resulted in their widespread use especially in medicine [16]. The extensive integration of AI into our daily lives has created the need for evaluating its performance in terms of quality, credibility, accuracy and comprehensibility [17]. Even though generative AI tools offer users apparently acceptable and satisfying answers, the data generated by them are not always enriched by evidence-based practice which may lead to misinformation and confusion among the patients [18]. Therefore, overreliance on AI tool as a standalone source of medical information may escalate the risks incidental to misinformation in medical contexts [17].

Scabies is an important health issue which requires prompt diagnosis and treatment in order to prevent transmission and long-term complications [19]. A study from Türkiye which was conducted by Atalık et al. [20] found the incidence rate of scabies to be 0.5–0.9% before the pandemic period and 3.1–4.4% in 2020–2022, which underlines the fact that scabies has a remarkable burden of disease in Türkiye. Additionally, another study from Türkiye pointed out the fact that the dermatological life quality index was moderately-to-severely affected in a vast majority of the patients and sleep disturbance associated with itching was a common problem [21]. Therefore, most patients may find it practical and straightforward to ask their most frequently raised concerns and queries about the diagnosis, pathogenesis and treatment of scabies to generative AI tools. In a recent study by Yılmaz et al. [9], a mobile application which utilizes a deep learning approach was shown to achieve high rates of sensitivity, accuracy and specificity in recognizing the clinical images of scabies. This study exemplifies how deep learning AI systems can be fostered to aid in the diagnosis and management of scabies cases, integration of cutting-edge AI technology into human clinical expertise will most likely result in the better healthcare results. Even though in the present study, we did not assess the performance of deep learning-based AI systems in the diagnosis of scabies-related clinical pictures, our study’s results also point out the fact that generative AI tools have the capacity to provide convincing and elucidative answers to the most frequently asked scabies-related questions.

In another recent study from Türkiye which analysed the videos related to scabies and scabies treatment on YouTube, 105 videos were evaluated and broadcasters were classified as news channels, healthcare professionals, healthcare websites, independent broadcasters and healthcare organizations [22]. The content of these videos were evaluated by using modified DISCERN, GQS, and the Journal of the American Medical Association (JAMA) scales in terms of accuracy, quality and credibility [22]. GQS, JAMA and modified DISCERN scores of the videos published by the healthcare practitioners were found to be higher when compared to the other videos uploaded by other broadcasters. The outcomes of the study once again underline the fact that the information delivered by the independent individuals is less trustworthy and may impose the risk of misinformation, thus it is concluded that healthcare professionals must undertake a more proactive role in social media platforms [22].

AI also has the capacity to generate scientific articles about a specific topic over a short period of time, which brings up the questions related to the integrity and credibility of the created scientific work [18]. AI hallucinations are referred as incorrect and fabricated outputs generated by AI tools [23,24]. Many forms of AI hallucinations may exist including referencing a non-existing scientific article or assimilating sources or statistics [18,25]. In a study by Májovský et al. [18] ChatGPT language model was used to compose a fraudulent scientific article about neurosurgery. After the article was created, it was reviewed for credibility and coherence by specialists [18]. The results showed that ChatGPT is capable of generating a highly persuasive deceptive scientific article, which included standard sections of a human-generated scientific article [18]. However, when the reference list was checked, 8 references out of 17 references were found to be defective (non-existing, contently incorrect) [18]. Another study by Walters et al. [23] used ChatGPT-3.5 and ChatGPT-4 to compose mini literature review articles on 42 multidisciplinary subjects. After the article was generated, the prevalence of the fabricated references was determined: 55% of the citations provided by ChatGPT-3.5 and 18% of the citations provided by ChatGPT-4 were found to be fabricated [23]. In our study, ChatGPT-5.2 provided references across all scabies-related questions without any hallucination however DeepSeek’s hallucination rates ranged from 16.7% and 20.0%. Additionally, DeepSeek demonstrated statistically significantly higher hallucination rates in comparison to both Claude Sonnet 4.5 and ChatGPT-5.2. In concordance with the results in the literature, our study once again highlights that hallucination rates may vary across different large language models; AI-generated data should therefore be scrutinized before processing and using in scientific purposes.

One key finding from this study is that high performance scores—particularly in accuracy and quality—can occur alongside higher rates of citation hallucinations, especially in models such as DeepSeek. This suggests that a model’s ability to generate strong content does not automatically make it reliable when it comes to referencing sources. A possible explanation is that some models may produce responses that appear coherent and well-informed while at the same time generating citations that are fabricated or impossible to verify. This finding points to a distinction between informational quality and source credibility, and it underscores the need for caution when interpreting AI-generated references in clinical and patient education settings.

The use of artificial intelligence in delivering health information also raises important ethical concerns. Users may perceive AI-generated responses as authoritative, even when those responses contain inaccuracies or misleading content [17,18]. This creates risks such as inappropriate self-diagnosis, delayed medical consultation, and unsafe self-management practices [18,23]. For these reasons, transparency, accountability, and clear communication about the limitations of AI are essential when integrating such tools into patient education. In this context, human oversight remains necessary to reduce potential harms and to support safe, informed health-related decision-making.

On a practical level, the safe integration of AI into patient education requires several key measures. These include maintaining human oversight, verifying the accuracy of AI-generated information, clearly communicating the system’s limitations to users, and putting quality control mechanisms in place to reduce the risk of misinformation.

### Limitations

The extent of the present study was limited only to scabies, hence the outcomes of the present study may not be generalized to other cutaneous or systemic diseases. Since large language models are being updated regularly via new training data, the data generated by them are likely to change over time. Additionally, the evaluators’ subjectiveness in assessing the accuracy, reliability and quality of the answers might have had a considerable impact upon the scores. Reliability, quality, and accuracy assessments were performed by a single dermatologist, which may introduce evaluator-related bias.

## 5. Conclusions

Patient education plays a critical role not only in the clinical management of dermatological conditions but also in preventing disease transmission within the community, particularly for highly contagious infections such as scabies. Inadequate or inaccurate health information can lead to delays in diagnosis, inappropriate self-treatment, sustained transmission within households, and an increased public health burden.

Conversational artificial intelligence technologies have the potential to serve as scalable tools for patient education, especially in settings where access to healthcare professionals is limited.

In this study, DeepSeek demonstrated higher accuracy and overall quality but also exhibited a greater frequency of hallucinations. In contrast, ChatGPT-5.2 showed better readability and reliability. Overall, these findings suggest that while large language models differ across various dimensions—including quality, readability, accuracy, and reliability—they are capable of producing clinically relevant but variable-quality information.

Given their increasing accessibility and potential for widespread use, AI-based chatbots are likely to play a growing role in patient information seeking. Nevertheless, variability in their performance and the presence of misleading or potentially unsafe content underscore the need for continuous evaluation and oversight by healthcare professionals. Inaccurate or unsafe guidance, in particular, may lead to inappropriate self-management decisions and possible adverse outcomes, especially since users often perceive AI-generated responses as authoritative.

Accordingly, these tools should be positioned as supportive educational aids rather than as replacements for professional medical consultation. Strengthening quality control mechanisms and improving the safety and reliability of responses remain essential before integrating such tools into broader public health strategies.

Future research should extend beyond content evaluation to examine user behavior and real-world impact. Studies could explore how patients interpret and act upon AI-generated responses, particularly in cases involving potentially unsafe or misleading recommendations. Furthermore, future studies should incorporate larger and more diverse datasets, including real-world user queries from multiple platforms, to enhance generalizability and better reflect actual patient information needs.

## Figures and Tables

**Table 1:** Comparative performance of AI models across evaluation metrics.

Model	mDISCERN (Mean ± SD)	GQS (Mean ± SD)	Flesch Reading Ease Score (Mean ± SD)	Accuracy Assessment (Mean ± SD)
Claude Sonnet 4.5	28.70 ± 4.16	4.10 ± 0.45	49.75 ± 10.93	4.35 ± 0.49
ChatGPT-5.2	33.60 ± 1.79	4.45 ± 0.51	63.25 ± 11.50	4.60 ± 0.50
DeepSeek	32.10 ± 2.13	5.00 ± 0.00	51.30 ± 8.14	5.00 ± 0.00

## Data Availability

The data supporting the findings of this study are available in the Supplementary Materials. Further inquiries can be directed to the corresponding author.

## Supplementary Materials

The following supporting information can be downloaded at: https://www.mdpi.com/article/10.3390/healthcare14101278/s1. Questions and responses used for evaluating AI chatbot performance. Hallucination data identified in AI-generated responses.
