# Supplementary material for: Can Artificial Intelligence Support Patient Education in Scabies? A Comparative Analysis of Large Language Model Responses
Source: Healthcare (Basel). 2026 May 8;14(10):1278. doi: 10.3390/healthcare14101278 (PMC13206425; doi:10.3390/healthcare14101278)
Supplement: Supplementary file 1 [file healthcare-14-01278-s001.zip › healthcare-4236724-supplementary.pdf]

Questions	Claude Sonnet 4.5 Hallucinated References (n)	Claude Sonnet 4.5 Valid References (n)	Claude Sonnet 4.5 Hallucination Rate (%)	ChatGPT5.2 Hallucinated References (n)
1	1	10	9.1	0
2	1	11	8.3	0
3	0	7	0	0
4	2	9	18.2	0
5	1	9	10	0
6	-	-	-	0
7	-	-	-	0
8	-	-	-	0
9	-	-	-	0
10	-	-	-	0
11	-	-	-	0
12	-	-	-	0
13	1	13	7.1	0
14	0	11	0	0
15	-	-	-	0
16	2	6	25	0
17	-	-	-	0
18	0	11	0	0
19	-	-	-	0
20	1	10	9.1	0

ChatGPT5.2 Valid References (n)	ChatGPT5.2 Hallucination Rate (%)	DeepSeek Hallucinated References (n)	DeepSeek Valid References (n)	DeepSeek Hallucination Rate (%)
6	0	0	6	0
5	0	0	4	0
7	0	0	5	0
6	0	0	4	0
5	0	0	4	0
7	0	0	5	0
5	0	0	5	0
5	0	0	4	0
5	0	-	-	-
5	0	0	4	0
7	0	1	4	20
6	0	0	5	0
6	0	0	5	0
5	0	0	4	0
6	0	0	5	0
7	0	1	5	16.7
6	0	0	4	0
6	0	0	4	0
7	0	-	-	-
7	0	-	-	-
